# A Survey of Keystroke Dynamics Biometrics

**DOI:** 10.1155/2013/408280

**Published:** 2013-11-03

**Authors:** Pin Shen Teh, Andrew Beng Jin Teoh, Shigang Yue

**Affiliations:** ^1^School of Computer Science, University of Lincoln, LN6 7TS, UK; ^2^School of Electrical and Electronic Engineering, Yonsei University, Seoul 120-749, Republic of Korea; ^3^Predictive Intelligence Research Cluster, Sunway University, Bandar Sunway, 46150 P.J. Selangor, Malaysia

## Abstract

Research on keystroke dynamics biometrics has been increasing, especially in the last decade. The main motivation behind this effort is due to the fact that keystroke dynamics biometrics is economical and can be easily integrated into the existing computer security systems with minimal alteration and user intervention. Numerous studies have been conducted in terms of data acquisition devices, feature representations, classification methods, experimental protocols, and evaluations. However, an up-to-date extensive survey and evaluation is not yet available. The objective of this paper is to provide an insightful survey and comparison on keystroke dynamics biometrics research performed throughout the last three decades, as well as offering suggestions and possible future research directions.

## 1. Introduction

Technology development over the past decade has contributed to the escalating access and storage of confidential information in digital devices. Therefore, the need for a more secure authentication mechanism becomes imminent. 

### 1.1. Types of Authentication

Authentication in short is the process of verifying a person's legitimate right prior to the release of secure resources. Generally this is achieved by counterchecking unique information provided by an individual. This information can be broadly subdivided into three categories namely *knowledge*, *token,* and *biometrics*-based authentication as summarized in Table 1 and discussed as follow.

#### 1.1.1. Knowledge

*Knowledge* commonly regard as something a person knows [1], which generally resides in the form of texture or graphical password, personal identification number (PIN), and pattern code. Password-based authentication has been an established method for access control in variety of systems since the past three decades [2]. Cost effectiveness and simple implementation have been the forefront reasons for the continuous dominance of password. Nevertheless, the ability for it to provide confident and secure authentication has been wearing, due to reasons such as the wrongful use of password and increased intrusion attacks. Simple password is the primary choice when it comes to password selection, such as date of birth, nickname, initials, and regular dictionary words that is either easily guessed or hacked. To aggravate the situation, users always tend to use the same or similar password for multiple systems. These bad usage habits contribute to the deterioration of knowledge-based authentication quality.

#### 1.1.2. Token

*Token* refers to an object that requires user to physically possess as a form of authentication. Common tokens include but not limited to swipe cards, credit cards, and minidevices. Although large-scale deployment is relatively simple [3], it comes with its own weakness. Token are vulnerable to loss or theft as user may find it inconvenient or difficult to keep it safe at all times. This implies that there is no assurance on uniquely identifying a legitimate user even with the ownership of token. Typically this shortcoming can be resolved by using token alongside knowledge-based method. At such, these two entities together render a simple two-factor authentication process that produces a stronger authentication based on the assumption that the secrecy of knowledge is not breached.

#### 1.1.3. Biometrics

*Biometrics* refers to certain physiological or behavioral characteristic that is uniquely associated to a person. This trait is highly distinctive and can be utilized for distinguishing different individuals. 

Physiological biometrics refers to a person's physical attribute, such as fingerprint, face, and iris. It is well known for its permanence and high uniqueness that promote high recognition accuracy. Unfortunately, it is not likely to be revoked if compromised (unable to change fingerprint pattern) [4], may possibly suffer low public acceptance due to invasiveness (iris scanning), and could be unlikely practical in large-scale deployment due to implementation cost (DNA analysis).

The way people do things such as speaking (voice), writing (signature), typing (keystroke dynamics), and walking style (gait recognition) are known as behavioral biometrics. Behavioral biometrics has the edge over its physiological counterpart on the ability to work in stealth mode verification. As such, minimal interaction is required during authentication process reduces invasiveness and thus promotes user acceptability. In addition, in the event if one's behavioral attribute is compromised, it is likely to be replaced (changing to a new password, thus, new keystroke print or new written signature) [5]. While these merits may be encouraging, they are normally inferior to physiological biometrics in terms of variability (voice changes along with aging factor) and may consequently influence verification accuracy.

### 1.2. Objective

Our objectives and contributions of this paper are listed as follows.

1. Present a comprehensive survey with the inclusion of most recent research papers up to year 2012 covering a total of 187 publications in the form of journal, conference proceeding, thesis, patent, and white paper.
2. Compliment neglected information in earlier reviews [6–8], such as data acquisition methods, experimental settings, template retraining, outlier handling, and feature quality control.
3. Lower the entry barrier to this field by providing a comprehensive reference for novices.
4. Offer a wide range of comparisons in diverse angles and perspectives in terms of experimental protocol evaluation, classifier categorization, and result comparison organization.
5. Recommend potential opportunity for enhancement and exploitation.

There exist a few review publications [6–8], specifically in the domain of keystroke dynamics as shown in Table 2. They vary in terms of year of publication covered, scope of discussion, length and depth of review, comparison methodology, opinions, remarks, and suggestions of potential area for future exploitation.

### 1.3. Organization

The organization of this paper is structured as follow: Section 2 covers the overview, advantages, disadvantages, and evaluation criteria of keystroke dynamics authentication system. Whereas Section 3, reveals various experimental platform and protocol followed by an in depth look into different data acquisition procedures used by fellow researchers in Section 4. The comparison on feature data used and methodology will be examined in Sections 5 and 6, respectively, while the experimental comparison and result will be shown in Section 7. Finally, Section 8 concludes the review with our recommendation and potential research opportunity.

## 2. Keystroke Dynamics

Keystroke dynamics refers to the process of measuring and assessing human's typing rhythm on digital devices. Such device, to name a few, usually refers to a computer keyboard, mobile phone, or touch screen panel. A form of digital footprint is created upon human interaction with these devices. These signatures are believed to be rich in cognitive qualities [9], which is fairly unique to each individual and holds huge potential as personal identifier.

### 2.1. Overview

**Figure 1:**
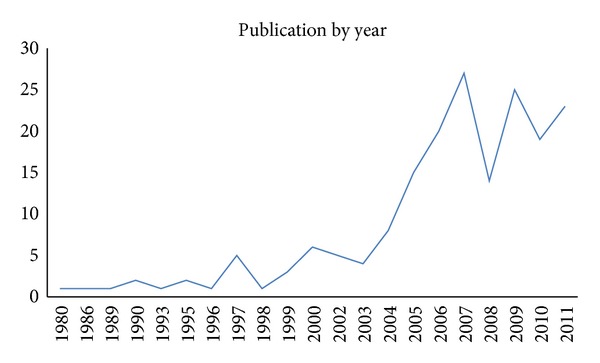
Graph clearly indicates an increasing trend on research work conducted on keystroke dynamics domain.

**Figure 2:**
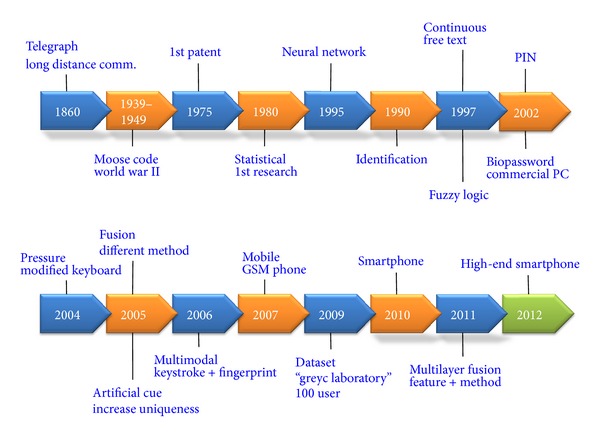
A general timeline on the overview of keystroke research work evolution.

The emergence of keystroke dynamics biometrics was dated back in the late 19th century, where telegraph revolution was at its peak [10]. It was the major long distance communication instrument in that era. Telegraph operators could seamlessly distinguish each other by merely listening to the tapping rhythm of dots and dashes. While telegraph key served as an input device in those days, likewise, computer keyboard, mobile keypad, and touch screen are common input devices in the 21st century. Furthermore, it has been noted that keystroke pattern has the same neurophysiologic factors that make hand written signature unique [11], where humans have relied on to verify identity of an individual for many centuries. In fact, keystroke pattern is capable of providing even more unique feature for authentication, which includes key press duration and latencies, typing rate, and typing pressure. Among the earliest significant keystroke dynamics research work on authentication was conducted by [12], ever since, this domain has gradually gained momentum (Figure 1).  Figure 2 shows the timeline development in the area of keystroke dynamics biometrics, which will be discussed throughout the paper.

### 2.2. Advantages

#### 2.2.1. Uniqueness

Keystroke event can be measured up to milliseconds precision by software [13]. Thus, it is impractical to replicate one's keystroke pattern at such high resolution without enormous amounts of effort.

#### 2.2.2. Low Implementation and Deployment Cost

In contrast to traditional physiological biometric systems such as palm print, iris, and fingerprint recognition that rely on dedicated device and hardware infrastructure, keystroke dynamics recognition is entirely software implementable. The benefit of low dependency on specialized hardware not only can significantly reduce deployment cost but also creates an ideal scenario for implementation in remote authentication environment.

#### 2.2.3. Transparency and Noninvasiveness

One of the significant edge keystroke dynamics biometrics has over other options is the degree of transparency it provides. It requires none or minimal alteration to user behavior since the capture of keystroke pattern is done via backend software implementation. In most cases, user might not be even aware that they are protected by an extra layer of authentication. This simplicity not only considerably favors system designer but also to those end user with little or no technical background.

#### 2.2.4. Increase Password Strength and Lifespan

Password has been the most widely deployed identity authentication methods despite the systems that rely solely on single credential set constitute weakness and vulnerability. Researchers have identified keystroke dynamics biometrics as a probable solution that is able to at least add an extra layer of protection and increasing the lifespan of password. Keystroke dynamics biometrics provide the capability to fuse the simplicity of password scheme with increased reliability associated with biometrics. By using keystroke dynamics biometrics, user can focus on creating a strong password whilst avoid being overwhelm by different sets of password.

#### 2.2.5. Replication Prevention and Additional Security

Keystroke patterns are harder to be reproduced than written signatures. This is because most security systems only allow limited number of erroneous input attempts before locking down the account. Additionally, integration of keystroke dynamics biometrics leaves random password guessing attack obsolete [14], and stolen credentials become entirely insignificant, since successful possession of secret key is only a mere condition of the entire authentication chain. Even if it does get compromised, a new typing biometric template can be regenerated easily by choosing a new password.

#### 2.2.6. Continuous Monitoring and Authentication

Continuous monitoring and authentication have often been sidelined yet they are relatively important. Keystroke dynamics biometrics offer a way to continuously validate [15] the legitimate identity of a user. As long as user interaction with the system through input devices persists, keystroke pattern can be constantly monitored and reevaluated.

### 2.3. Disadvantages

#### 2.3.1. Lower Accuracy

Keystroke dynamics biometrics are inferior in terms of authentication accuracy due to the variations in typing rhythm that caused by external factors such as injury, fatigue, or distraction. Nevertheless, other biometric systems are not spared by such factors either [16].

#### 2.3.2. Lower Permanence

Most behavioral biometrics generally experience lower permanency compared to physiological biometrics. Typing pattern of a human may gradually change following the accustomization towards a password, maturing typing proficiency, adaptation to input devices, and other environmental factors. However, researchers have recommended methods to constantly update stored keystroke profile [17–19] that may resolve this issue.

### 2.4. Keystroke Dynamics System Overview

A typical keystroke dynamic authentication system consists of several components, namely, data acquisition, feature extraction, classification/matching, decision, and retraining.

#### 2.4.1. Data Acquisition

This is the fundamental stage whereby raw keystroke data are collected via various input devices. These may consist of normal computer keyboard [20–22], customized pressure sensitive keyboard [21, 23], virtual keyboard [24], special purpose num-pad [25–27], cellular phone [28, 29], and smart phone [30].

#### 2.4.2. Feature Extraction

Raw keystroke data are then processed and stored as reference template for future usage. Some preprocessing procedures may be applied before feature extraction to ensure or to increase the quality of feature data. These steps may include feature selection [31], dimension reduction [32], and outlier detection [33–35].

#### 2.4.3. Classification/Matching

The essence of most recognition systems falls in this phase, where feature data are categorized and discriminated for later use to make decision. Vast amount of diverse algorithms have been applied by previous researches with a common goal of increasing authentication accuracy. Majority of the pattern recognition algorithms employed in the literature for the past three decades can be broadly classified into two main categories, namely, statistical and machine learning approaches. Further discussion of the methods is dedicated at later section.

#### 2.4.4. Decision

Claimant's feature data is presented to the system and compared to the reference template via classification algorithms. A final decision will be made based upon the outcome of classification or matching algorithm to determine if a user is legitimate or otherwise. Prior to decision making, fusion strategy [3, 36, 37] may be applied to strengthen authentication accuracy.

#### 2.4.5. Retraining

As discussed earlier due to the variability of user typing pattern, it is therefore necessary to constantly renew the stored reference template to reflect the ongoing changes. Several researchers have proposed diverse adaption mechanisms [38, 39] with regard to this issue.

### 2.5. System Evaluation Criteria

The effectiveness of a keystroke dynamics authentication system is usually gauged by the recognition rate of the system. However, in order to put forward this technology into real world practice, equal weights should be put in consideration on several other essential criteria [40] as shown below. 

#### 2.5.1. Effectiveness

Effectiveness indicates the ability of a method to correctly differentiate genuine and imposter. Performance indicators employed by the researches are summarized as follow.

*False Rejection Rate* (FRR) refers to the percentage ratio between falsely denied genuine users against the total number of genuine users accessing the system. Occasionally known as *False Nonmatch Rate* (FNMR) [19] or *type 1 error* [9]. A lower FRR implies less rejection and easier access by genuine user.

*False Acceptance Rate* (FAR) is defined as the percentage ratio between falsely accepted unauthorized users against the total number of imposters accessing the system. Terms such as *False Match Rate* (FMR) [19] or *type 2 error* [41] refers to the same meaning. A smaller FAR indicates less imposter accepted. 

*Equal Error Rate* (EER) is used to determine the overall accuracy as well as a comparative measurement against other systems. It may be sometimes referred to as *Crossover Error Rate* (CER) [42]. Result comparison portrayed in the next section will mainly be express with FAR, FRR, and EER.

#### 2.5.2. Efficiency

The efficiency refers to the complexity of method employed, which normally considered better if complexity is lower. A computationally expensive method does not only put mounted strain to hardware but also frustrates user with longer waiting time.

#### 2.5.3. Adaptability and Robustness

Adaptability implies the ability of a system to accommodate gradual typing changes of user across time. Robustness indicates the capability to work well with users from diverse professions with dissimilar typing proficiencies.

#### 2.5.4. Convenience

This is an important factor that is directly related to user acceptability to the technology. The technology should offer user as much comfortable and transparency as possible by not overloading user with long inputs, memorization of complex strings, or provide huge amounts of repetitive input.

## 3. Experimental Setup and Protocol

### 3.1. Keystroke Dynamic Acquisition Device

#### 3.1.1. Normal Hardware

 One of the prime benefits of keystroke dynamics biometrics is low dependency on dedicated hardware infrastructure. For that reason, it is self-explanatory why most researchers go for readily available hardware for study. The most common choice is the widely available QWERTY keyboard [43, 44], followed by built-in laptop keyboard [45, 46]. 

Some research works, unlike others, only used specific portion of a hardware [47]. The research restricted user to use num-pad of a keyboard with just one finger to replicate an impoverished experimental condition. They believed that if good result was achieved in such simplistic provision, then implementation in a less restrictive environment could likely accomplish better performance.

On the other hand, [48] utilized Synaptic Touchpad attached to a notebook to measure finger pressure and position. Their intention was to implement keystroke dynamics biometrics on touch screen mobile devices, but due to the technology bottleneck at that point of time, it is understood why a cheaper alternative had been chosen. Although the device sensitivity might not be anywhere comparable to a real touch screen technology, the idea was inspirational for researchers when the technology becomes available.

#### 3.1.2. Customized Hardware

Conventional input devices such as normal computer keyboards are only capable of producing keystroke timing vector as feature data for analysis. A secondary feature data that may be proven more distinctive is the pressure sequence while interacting with the input devices. Therefore, numerous researchers have tried to modify the existing devices [49–52] to include pressure sensitive receivers.

Another modification was made to a rubber membrane keypad that resembles an ATM machine input alignment [26], with the objective of improving security on a numeric PIN system. The original mounted printed circuit board underneath the keypad was replaced by custom fabricated force sensitive resistors. However, the actual implementation to the banking sector is rather doubtful due to the cost of replacement to the entire hardware infrastructure.

Leberknight et al. [27] pointed out that leveraging the effects of soft and hard key presses was crucial yet challenging for tailored made pressure sensitive devices. Parasitic capacitive coupling that occurs in over sensitive devices might distort feature quality. This raised the concern that a minimal benchmark on the accuracy of pressure input devices might be required if it is to be used in large-scale applications. However, we foresee that in the post-pc era [53], pressure sensitivity standards in personal digital devices will be able to meet the practical needs.

#### 3.1.3. Mobile Devices

While typographical input from computer keyboard has been the main focus at the infancy stage of keystroke dynamics research, numerical base input from portable communicational devices has gradually gained attention since the wide spread use of cellular phone globally in the 20th century [54].

Research works such as [28, 29] performed experiments on conventional numerical key pad cellular phone in attempt to authenticate user via short input text. The initiative was encouraging but the issue of cross-platform compatibility across diverse model of devices remains an open question. 

Along with the rapid evolution of technology, mobile devices have also gained greater processing capability. Java enabled Symbian phone was selected by [55] as the platform for their study. They attempted to use several computational expensive neural network algorithms for recognition and have yielded some encouraging results. Unfortunately, a major setback was the degradation of response time to the mobile device that might affect user acceptance.

A more recent publication reported by [30] used early generation smart phone with touch sensitive screen, which could be interacted via finger or stylus (special pointing stick). The trend of applying keystroke dynamics biometrics to newer hardware technology should be encouraged, since the interaction method, processing capability, and availability of these devices open to new research dimension and opportunity.

#### 3.1.4. Other Hardware

Although keyboard, num-pad and mobile phone have been the dominating input devices for keystroke dynamics research, some works have also been performed on less common equipment. For instance, four pairs of infrared proximity-sensing devices were used to project a virtual numeric keyboard on a piece of white surface [24]. In the experiment, user's finger has to be held at a 90 degree angle to the surface keyboard for proper detection. Therefore, with the increase complication of input procedure, the usability has been a cause of doubt. Conversely, [56] implemented a more practical multimodal authentication by combining keystroke dynamics input and fingerprint by using a portable point of sales device.

### 3.2. Device Freedom and Experimental Control

Device freedom refers to whether the equipment used in the experiments is standardized or the users have the flexibility to use their own input devices. Among approximately 187 publications surveyed, 34% used predefine standard device, 17% performed experiment on user's own device, while the remaining 49% were unknown due to inadequate information. However, it is reasonable to assume that they employ fixed devices strategy since those experiments that allow user to make use of their own devices often mentioned explicitly.

The fixed setting can get rid of introducing uncontrollable variables such as device familiarity, device compatibility, and functional differences hence, the result is solely reflected by the discriminative power of keystroke dynamics feature or classification algorithm [33, 57]. The rationale behind this thought is that the user may be more accustomed to their own input devices that may lead to distortion of experimental data. Although some may not clearly state this information, it is no doubt that experiments that use customized devices (e.g., pressure sensitive keyboard) were provided by the researchers. This might be the reason why it is in favor by the most researchers, almost twice the amount compared to user centric devices.

In contrast, some research works employed different approaches by not restricting the usage of device. For instance, [58] requested user to download an executable program into their own personal computer for data collection, while [59] implemented a JavaScript web-based client, where users are free to use any machine as long as it comes with a web browser. At such, it can be argued that the experimental results obtained closely resembled real world scenario.

Another vital variable is the constraints that researchers imposed particularly in data collection phase. Experiments may be conducted entirely in a supervised environment with a strict protocol such as in [25]. Video clips of legitimate user login trials are prerecorded and later presented to the imposter in an attempt to imitate genuine user login during testing stage. Apart from that, experiments that involved additional customized hardware [21] or software library [33] will apparently be best to be performed under controlled laboratory environment. At such, the hassle and complexity of experimental deployment as well as the cost of implementation can be kept minimal. It was also argued by [47] that one of the benefits of operating experiments under stringent protocol is to single out external factor from inflicting noise. As a result, primary experimental variables could be clearly evaluated [60]. However, there may be a concern that the result obtained under such control setting may not reflect real world scenario.

On the contrary, experiments that did not impose restriction or unmonitored offered user comfort and flexibility that resembled realistic condition. As an example, the nature of the experiment conducted by [59] required the collection of typing pattern of user daily activity on a computer. Data collected by allowing user to use their preferential device is more desirable than requiring user to work on an entirely unfamiliar device. Since lacking of constraints, the quality of data collected could be distorted or tempered with. Perhaps these might be the reasons why most research works perform under close administration, more than double of the amount of those uncontrolled.

### 3.3. Development Platform

Since the most common user interaction involving text and numerical input is through a personal computer, researchers who were working on keystroke dynamics are almost all based on local computer platform. Before the 21st century, keystroke dynamics experiment prototype was developed on operating system (OS) platform using third-generation programming language (3GL) such as FORTRAN [61] and Turbo Pascal [1]. Later when Microsoft products dominate most operating system, an experimental prototype was built on top of MS DOS [62] and windows environment [43] by using languages such as C++ [63] and Visual Basic [64]. 

Owing to the pace of internet development in the last decade, experimental platform has been shifted to the web-based environment [15] with web programming tools such as JavaScript [65], Java Applet [66], and Flash [67]. It is only in the last couple of years; several works have been developed based on mobile device environment. Starting off with mobile emulator [68], Symbian operating system [55], and most recently Android platform [30]. The association of development platform with keystroke dynamics research works in the literature can be summarized as OS (44%), web (17%), mobile (5%), and unknown (34%).

### 3.4. Authentication Protocol

#### 3.4.1. Verification versus Identification

Keystroke dynamics authentication can be categorized as *verification* and *identification*. *Verification* refers to the process of proofing a validity of claimed identity. In other words, “*is this person really who he or she declares to be.*” This is a one-to-one comparison procedure that required minimal overhead and is the most common scenario in our society's security access control environment. On the contrary, *identification* denotes “*is this person in our database, if yes, to whom this presented identity belongs to.*” Identification is generally more time consuming, slower in responsiveness, and require higher processing capacity. Nevertheless, identification mode has its own unique usage such as forensic investigation and intrusion detection.

Majority of keystroke dynamics research works have been investigated in the form of verification mode (89%) compared to identification (5%). Note that the remaining unknown (6%) authentication mode can be assumed to be verification, due to the fact that most researchers will mention in specific if their experiments involved identification mode.

#### 3.4.2. Static versus Dynamic

Keystroke dynamics coexist within two different modes of authentication. Static authentication mode attempts to verify user at the initial instance of user interaction with the system. These include the attempt of using keystroke dynamics biometrics to supplement password for security login [66, 69], physical access control [27], automated teller machine [70], and password sharing prevention [71]. 

Dynamic authentication mode deals with a different demand in computer security. The goal is to ensure that the authorized identity is still whom they claimed to be after initial login procedure. It is also referred to as continuous [1, 72] or reauthentication [73, 74] in the literature. The main advantage over static authentication is the ability to continuously ensure the validity of a legal user throughout the interaction period. It is also usually capable of working in silent mode, which will not cause any or minimal inconvenience to the user. Possible application may include online examination [15, 75] and account activity monitoring [76]. Dynamic authentication was also recommended by [59] to be used for password recovery and intrusion detection purposes. Although dynamic authentication has gained momentum in recent years, the number of researches is still evidently small (10%) compared to static authentication (83%). Among the probable reasons may be the complexity of experiment setup and less application as compared to static authentication.

## 4. Data Acquisition

Data acquisition is the preliminary and essential stage of keystroke dynamics research. Due to the lower maturity compared with other established biometrics, publicly available benchmark databases are limited. Although some researchers have taken the initiative to share their homemade data set, due to the diverse development setups and variables, many have chosen to generate in-house data set. Therefore, this section attempts to provide an overview on most of the properties of dataset employed.

### 4.1. Data Size

**Figure 3:**
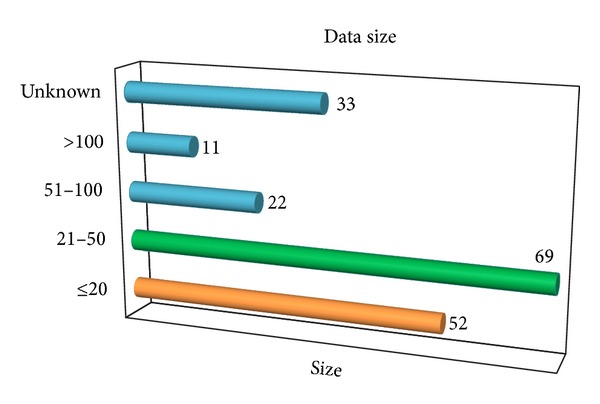
Frequency distribution of data size in keystroke dynamics experiments.

It is collectively agreed that experiments that includes large number of subjects better signify the scalability of study. Regrettably most of the studies performed involve only small number of subjects. This is understandable due to various issues and difficulties encountered in data collection process (to be discussed in the following section). Generally most research works involve less than 50 subjects, with a vast amount as low as 10 to 20 people. Although some research works reported to have involved large number of users (118 [77] and 250 [78] users), only a portion of the population completed the entire experimental cycle. A clear overview on the frequency distribution of data population has been summarized in Figure 3.

### 4.2. Subject Demographic

Most experimental subjects involve people around a researcher's institute ranging from undergraduate and postgraduate students [74], researchers [55], academicians, and supporting staffs [18, 76]. Although it may be argued that these populations may not be able to represent the global community, but it is still the primary option as it is the closest readily available resource. 

Even though several research works has claimed to involve population from broad age distribution (20 to 60) [55, 66, 79], emphasis should be placed on a more important aspect, such as the typing proficiency of these users. Apart from [12], where the whole population consists of skilled typists, others involved untrained typists who are familiar with the input device [80, 81]. However, none of the experiments specifically conducted on users that come from entirely low typing proficiency.

### 4.3. Data Type

**Figure 4:**
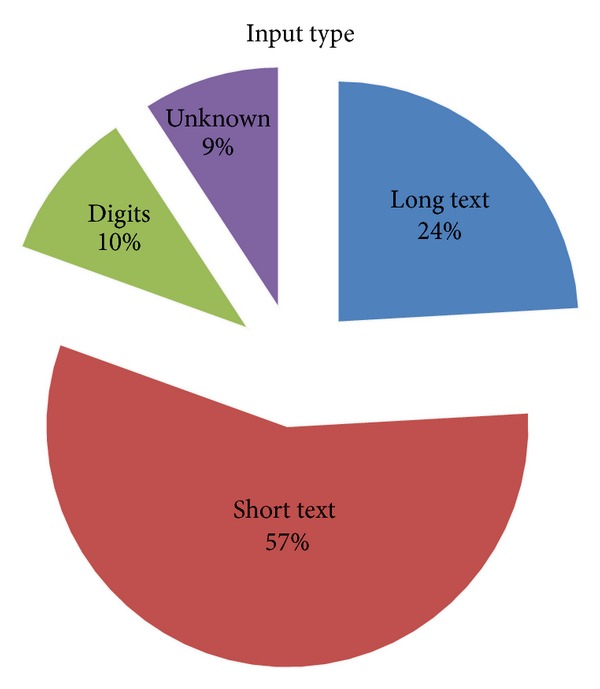
The percentage distribution of various types of input data.

In general, experimental subjects are required to either provide character-based text or purely numerical inputs [82]. The majority of research works with character-based inputs are illustrated in Figure 4. The input type can be further subdivided into long or short text. Short inputs normally consist of username [62, 83], password [84, 85], or text phrase [61, 86], while long inputs are usually referred to paragraphs of text enclosing 100 words or more [87, 88].

Freedom of input is another determinant factor that distinguishes keystroke dynamics research. The evaluation that requests experimental subject to type a predetermined input [89, 90] has the advantage of utilizing sample data from different users in the same database pool. This method significantly increases the number of imposter samples without the need of collecting them separately. On the other hand, an experiment that offers the flexibility of input data may require more efforts to collect additional test data [85, 91]. Having said that, user defined input resembles closer to real world scenario than fixed text. Furthermore, it is infeasible to constrain the input text in some cases such as [22, 72, 74], due to the nature and objective of the experiment where the user must have the freedom of input. Therefore, the number of research works on both types of inputs is fairly even.

### 4.4. Genuine and Imposter Samples

Data collected will eventually be used for performance evaluation. The most common way of performance measurement is the degree of accuracy of a system's ability to distinguish genuine and imposter. 

Imposter samples are usually obtained by either the same individual who contributes to the generation of genuine samples in database [92] or via another group of individuals attacking or simulating the genuine samples stored in the database [22]. The former imposes participants to provide more inputs and devote more time in the experiment. The lengthy process may deter volunteer participation. On the other hand, the latter required less participation effort by each user but a separate pool is required. Difficulty to secure large pool of users due to resource limitation may be the reason why only 38% of the experiments in the literature opt for this way as compared to the earlier at 46%, while the unknown stands 16%.

An alternative that may resolve this issue is by partitioning user sample data into two subsets. The first subset is used as training while the remaining as testing sample [41]. Leave-one-out, cross validation, or random split can be used in this context [87]. Having this way, separate imposter data collection set is supplementary. Although it seems to be advantageous, this method is only applicable if every subject's input is identical.

### 4.5. Input Repetition

**Figure 5:**
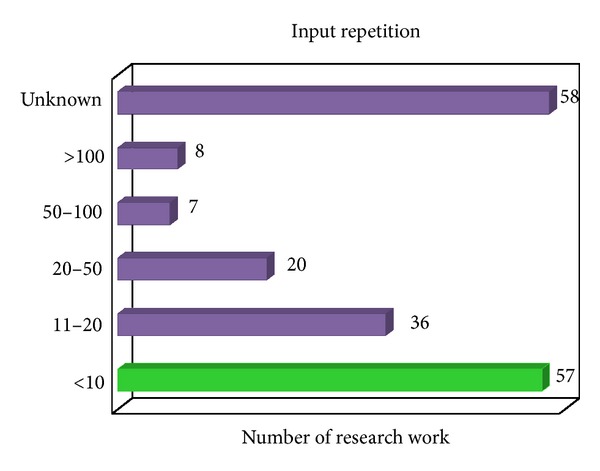
The categorized distribution on the number of input repetition in the keystroke literature.

In order to generate reference template, several instances of sample data are required. The greater amount of samples used in constructing reference template, the closer it resembles one's typing model [93] and recognition rate may also be potentially better as proven by [78, 94]. However, it is infeasible to collect large number of sample data during enrolment stage. Therefore, a balance should be struck while selecting the optimal number of sample repetition for an experiment. According to the trend in the literature, the benchmark was positioned at less than ten as shown in Figure 5. Nevertheless, sample collection can be divided into several sessions over a period of time, thereby not only reducing the initial load but also reflecting typing variability (further discussion will be given in the following section).

### 4.6. Sample Collection Interval

As discussed in the previous section, the greater number of samples collected the more accurate and conclusive a test result can be from statistical point of view. However, it is impractical to request huge amount of inputs from user at a single instance. More importantly keystroke dynamics are to behavioral biometrics where variability of typing is expected appear across different sittings [95]. Therefore, several sessions of data collection would ideally leverage one's typing evolution. 

In view of this, some researchers split the data collection phase into several different frequencies and interval separation length. These include a daily sitting over three weeks duration [21], three sessions within six days [96], or five sessions with one week apart [93]. Having said that, the majority data collected in keystroke dynamics literature were within one sitting (73%). Problems such as user availability and commitment for corresponding sessions might be a pullback factor for employing multiple session data collection.

### 4.7. Public Data Set

To the best of our knowledge, we are able to access three publically available data sets shared online [97–99]. Although they may not be comparable to benchmark data set of other biometrics modalities, however, full credit should be given on the attempt to share their resource with the community. Since data collection is not a straightforward task, by doing so, at least, entry level researcher may have a platform to work on. A simple comparison among the data set can be seen in Table 3.

## 5. Feature Selection

**Figure 6:**
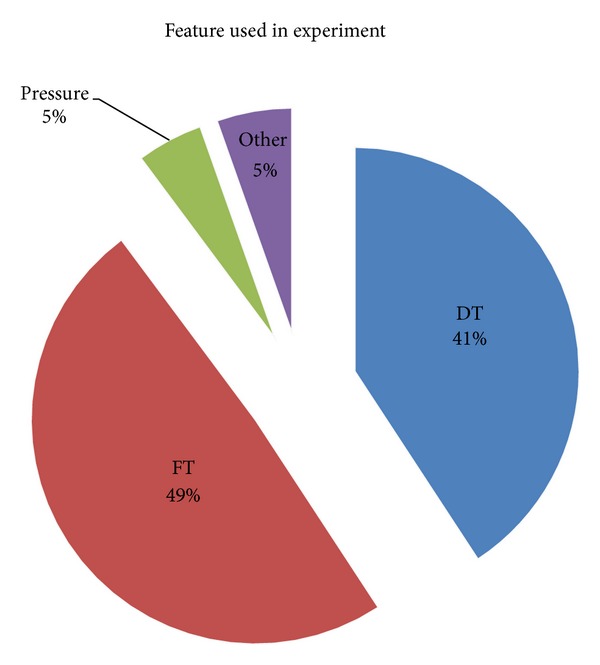
The percentage distribution of feature data extracted for keystroke experiment in the literature.

Keystroke dynamics biometrics are rich with distinctive feature information that can be used for recognition purposes. Among the easiest and common feature harvested by researchers is the timing measurement of individuals' keystroke inputs as shown in Figure 6.

Keystroke activity generates hardware interrupt that can be time stamped and measured up to microseconds (ms) precision [1]; therefore, it can be readily applied. In previous works, timing resolution of 0.1 s to 1 ms has been deemed to be sufficient [39]. By performing simple mathematical operation to these time stamp, timing duration, or interval between consecutive keystrokes can be obtained. 

Several attempts, although uncommon, of using keystroke pressure, typing speed [100], typing sequence difficulty [14], frequency of typing error [101], and sound of typing [102] have also been made. Due to the insignificant amount and unpopularity of the aforementioned feature type, the following subsections will focus on the discussion of the more popular timing feature. 

### 5.1. Di-Graph

Timing information of two consecutive keystrokes, better known as *di-graph*, is the major feature data represented in keystroke dynamics domain [72]. It is widely categorized into two types, namely, *Dwell Time* and *Flight Time*. Both are relatively equally weighted in terms of usage frequency among 187 research works as illustrated in Figure 6.

#### 5.1.1. Dwell Time (DT)

Dwell time refers to the amount of time between pressing and releasing a single key. In other words, how long a key was held pressing down. It is also worth noticing that several terms for DT appeared in the literature such as *duration time *[43, 84] and *hold time *[45, 103]. DT can be calculated by

(1) $$\begin{array}{c} \text{D}{\text{T}}_{n}=R_{n}-P_{n}, \end{array}$$

where *R* and *P* indicate the time stamp of release and press of a character, respectively, while *n* indicates the position of the intended DT. 

**Figure 7:**
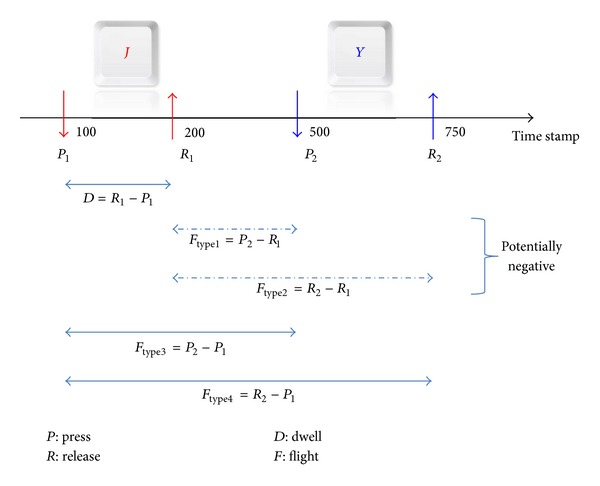
Figure depicts the different keystroke events of two characters “*J*” and “*Y*” along side with the formation of dwell time and flight time.

For instance, referring to Figure 7, DT for character “*J*” and “*Y*” is 100 (200–100) and 250 (750–500) correspondingly. The total number of timing vector of DT (*V*_DT_) that can be generated as follow:

(2) $$\begin{array}{c} V_{\text{DT}}=\left\{\text{D}{\text{T}}_{\text{1}},\text{D}{\text{T}}_{\text{2}},\text{D}{\text{T}}_{\text{3}},\text{…},\text{D}{\text{T}}_{s}\right\}, \end{array}$$

where *s* denotes the summation of characters in a string. In other words, the number of DT generated will always be the same as the length of a given string.

#### 5.1.2. Flight Time (FT)

Flight time refers to the amount of time between pressing and releasing two successive keys. It may also be termed as latency time [104, 105], interkey time [103, 106] or interval time [107, 108]. It always involves key event (press or release) from two keys, wich could be similar or different characters. FT may exist in four different forms as depicted in Figure 7. The formula to calculate each form are listed as follows:

(3) $$\begin{array}{c} \text{F}{\text{T}}_{\text{type1,}n}=P_{n+1}-R_{n},\\ \text{F}{\text{T}}_{\text{type2,}n}=R_{n+1}-R_{n},\\ \text{F}{\text{T}}_{\text{type3,}n}=P_{n+1}-P_{n},\\ \text{F}{\text{T}}_{\text{type4,}n}=R_{n+1}-P_{n}, \end{array}$$

where *R* and *P* indicate the time stamp of release and press of a character, respectively, while *n* indicates the position of the intended FT.

As an example FT_type1_ between character “*J*” and “*Y*” shown in Figure 7 is 300 (500–200), whereas the FT_type3_ is 400 (500–100). The previous literature pointed out the possibility of obtaining negative value (<0) for FT_type1_ [1, 109–111]. This situation occurs when an individual presses the next key before releasing the previous key. However, a closer observation shows that it is also possible for FT_type2_ to incur this property, albeit in a very exceptional circumstance. The total number of timing vector of FT (*V*_FT_) that can be generated is shown as follows:

(4) $$\begin{array}{c} V_{\text{FT}}=\left\{\text{F}{\text{T}}_{1},\text{F}{\text{T}}_{2},\text{F}{\text{T}}_{3},\ldots ,\text{F}{\text{T}}_{s-1}\right\}, \end{array}$$

where *s* denotes the summation of characters in a string. Differing from DT, the number of FT generated will always be one less than the length of a given string.

### 5.2. N-Graph

*N-graph* refers to the timing measurement between three or more consecutive keystroke events. It is better known as the elapse time between a key and the *n*th key event of a typing string. Despite many combinations of elapse time (ET), it can be extracted; the equation below is the most widely used when *n*-graph is concerned [91, 101, 112].

Consider

(5) $$\begin{array}{c} \text{E}{\text{T}}_{k}=P_{k+n}-P_{k}, \end{array}$$

where *P* indicates the time stamp of pressing a character, *n*  denotes *n*th number of graphs employed, while *k* represents position of the intended elapse time. The total number of timing vector of ET exists in *n*-graph which can be seen as follows:

(6) $$\begin{array}{c} V_{\text{ET}}=\left\{\text{E}{\text{T}}_{1},\text{E}{\text{T}}_{2},\text{E}{\text{T}}_{3},\ldots ,\text{E}{\text{T}}_{\text{s}-n+1}\right\}, \end{array}$$

where *s* denotes the summation of characters in a typing sequence.

From this survey, we noticed that 80% used di-graph; 7% used tri-graph; only 4% used *n*-graph, while 9% of the rest were unknown. The ability to generate significantly more instance of timing vectors could be the reason for the popularity of di-graph. As a result, any value of *n* that is greater than 3 (tri-graph) was rarely chosen except for the experiment that involved huge amount of input text [22, 81].

## 6. Methodology

### 6.1. Classification

Many classification methods have been applied in keystroke dynamics study over the last three decades. Keystroke dynamics recognition can be perceived as a pattern recognition problem and most of the popularly and commonly deployed methods can be broadly categorized as statistical (61%), machine learning approaches (37%), and others (2%).

#### 6.1.1. Statistical Approach

Statistical methods are the common choices not only at the infancy stage of keystroke dynamics research [12, 113, 114] but also in present work [65, 75, 115]. The popularity is directly related to the simplicity, ease of implementation, and low overhead. Among the common generic statistical measures include mean, median and standard deviation [57, 100, 116], statistical *t*-test [12], and *k*-nearest neighbor [24, 58, 73].

*Probabilistic* modeling is another variant of statistical approach that holds the assumption that each keystroke feature vector follows Gaussian distribution [20]. The main concept is that what is the likelihood of a given keystroke profile belonging to a particular class or individual who is registered in the database. Some widely used modeling techniques include Bayesian [45, 61, 96], Hidden Markov Model [82, 117, 118], Gaussian Density Function [18, 39, 108], and weighted probability [20, 56].

Meanwhile, *cluster analysis* is the technique of collecting similar characteristics pattern vectors together. The aim is to gather information about keystroke feature data in order to form a relatively homogeneous cluster [16]. Feature data categorized within a homogeneous cluster are very similar to each other but highly dissimilar to other clusters. K-mean [17, 31, 119] and fuzzy c-means [71] fall within this category.

**Figure 8:**
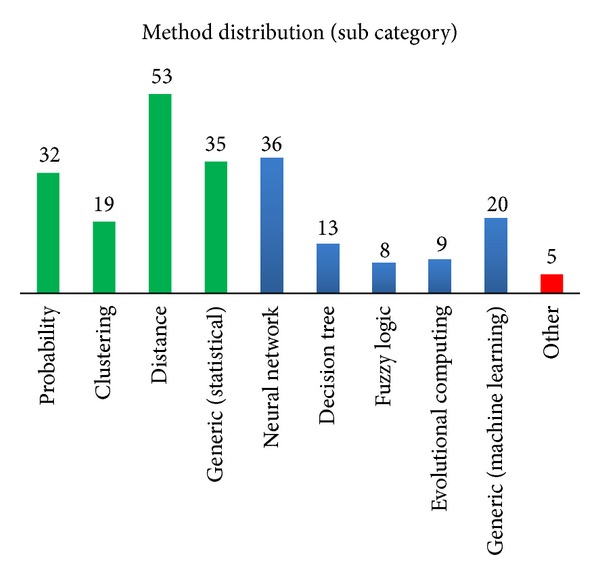
Percentage distribution among classification methods employed by keystroke research work.

The most popular method is simply by using *distance measure* as shown in Figure 8. In distance measure, the pattern of the claimant login attempt is calculated to determine the similarity/dissimilarity associated with a reference pattern in the database. Common measure used to compute distance score introduced in the literature included but is not limited to Euclidean [77, 120, 121], Manhattan [99, 122, 123], Bhattacharyya [81, 124], Mahalanobis [125], degree of disorder [43, 76, 126], and direction similarity measure [3].

#### 6.1.2. Machine Learning

Machine learning is widely used in the pattern recognition domain. The core idea is the ability to identify and classify pattern and make correct decision based on data provided. Subdomain under this category includes but not restricted to neural networks, decision tree, fuzzy logic, and evolutionary computing.

*Neural network* is a technique that mimics the biological neurons for information processing. Neural network is capable of providing an estimation of the parameters without precise knowledge of all contributing variables [86]. A classical neural network structure consists of an input layer, output layer, and at least one hidden layer [62]. Sample data is iteratively fed into the network to produce some outputs based on the current state of its initial predetermined weights. These outputs are compared to the true output, and an error value is computed. This value is then propagated backwards through the network so that the weights can be recalculated at each hidden layer to reduce the error value. The sequence is reiterated until the overall error value falls below a predefined threshold. 

Neural network is claimed to be capable of producing better result than the statistical methods [7]. However, the classifiers require not only genuine keystroke patterns but also intruders' to train the network. It may be impractical to obtain intruders' samples at the initial enrolment stage [127, 128]. Furthermore, any addition, removal or update on user profile in the system requires the whole network to be retrained and thus the amount of processing time increases. Database partitioning [20] and retraining during system idle period [55] has been suggested as an attempt to resolve this problem. Some widely used neural networks are radial basis function network [9, 49], learning vector quantization [62, 129], multilayer perceptron [24, 80, 86], and self-organizing map [130, 131].

*Decision tree* is a kind of learn by example pattern recognition technique that is suitable for classification problem involving small output class such as genuine or imposter. It is usually less computational intensive as compared to neural network [40]. The main concept is to recursively split training data so that the information gain ratio is maximized at each level of node in the tree. This step carries on until each node has only a single class example or information gain is exhausted [74]. Precaution should be taken to avoid over fitting the tree, which could lead to poor performance as well as high computational complexity. Some tree- based learning methods that are used in the literature were random forest [21, 33, 132] and J48 [74, 87].

*Fuzzy logic* uses multivalued logic to model problems with ambiguous data [14]. The key idea is to construct the boundaries of decision region based on training data with membership functions and fuzzy rules [55]. After the feature space has been identified, the degree of category in which a test template belongs to can be determined based on the computation of membership values. The instances of using fuzzy logic in keystroke dynamics authentication are [14, 23, 71]. 

*Evolutionary computing* has also been explored by researchers in hope to improve accuracy performance. Genetic algorithm [133, 134], particle swan optimization [135], and ant colony optimization [136] are the techniques that have been applied to select the most optimized keystroke feature for classification, thereby increasing classification accuracy.

Another renown classifier adopted by many studies [137–139], which distinguishes imposter patterns by creating a margin that separates normal patterns from imposters' is called *Support vector machine* (SVM). This method generates the smallest possible region that encircles the majority of feature data related to a particular class. SVM maps the input vector into a high-dimensional feature space via the kernel function (e.g. linear, polynomial, sigmoid, or radial basis function) [140]. The algorithm will then search for a function that encapsulates the majority of patterns contained in the input vector and vector outside this region. As a result, the separating function is able to create more complex boundaries and to better determine which side of feature space a new pattern belongs. SVM is claimed to have a competitive performance as compared to neural network and yet less computational intense [111]; however, the performance is questionable when the feature set is too large [38].

### 6.2. Retraining Module

Keystroke dynamics biometrics are behavioural traits, which implie that it is impossible to acquire an exact typing pattern of even from the same individual. This is useful for authentication, whereby the distinctiveness can be used to differentiate one's keystroke dynamics from anothers. On the other hand, it may also cause problems due to intraclass variability. A solution is needed to compensate the changes of legitimate user's gradual typing cadence over time.

Retraining refers to the recapture and refinement of users' biometric template upon successful verification of their biometric credential [141]. It is also known *as incremental learning procedure* [142], *template update mechanism* [19], and *adaptive module* [93]. If keystroke template remains unaccustomed to the gradually shift of typing pattern over time, system accuracy will be degraded over time. According to [19], 50% of improvement can be gained by having this module in place. However, the number of research works that engaged with retraining module is limited to only less than 20% among 187 literatures studied. 

#### 6.2.1. Growing Window

This method was alternatively known as progressive mode by [96]. The idea behind this technique is to append the latest user sample to their existing reference template. By doing so, the size of reference template may be increasing indefinitely, which may cause storage overhead. However, some algorithms employed may be spared or adjusted to avoid this consequence. For example, [18] utilized an alternative version of mean and standard deviation to avoid storing the entire preceding keystroke timing values. Nevertheless, the implementation of growing window is better than no adaptation at all [19].

#### 6.2.2. Moving Window

As oppose to growing window, moving window adds the new user sample to template profile and subsequently releasing the earliest sample, thereby retaining a constant template size. It is also known as *adaptive mode *[96] or *sliding window *[123]. A fixed window size is normally used, which is considered to be a disadvantage [17]. Despite the shortcoming, it is considered as an improved version of growing window [19]. It is interesting to investigate if window size correlates with system accuracy or what is the optimal length of window size to achieve best performance.

#### 6.2.3. Intelligent Mode

Intelligent mode is the combination of progressive (growing window) and adaptive mode (moving window) [89]. If the number of training vectors accumulated to a predetermined length, adaptive mode is used; otherwise, progressive mode will be deployed. Claimant vectors are only added if they do not differ significantly from the model. Experimental result shows that intelligent mode generally achieve better performance than the other two counterparts. 

#### 6.2.4. Retraining with Imposter Pattern

The methods discussed by far only involve retraining template with genuine authenticated samples. Dissimilarly, imposter samples were used in retraining process [129]. They claimed that by taking novel pattern into consideration, it could help the algorithm to exclude patterns that were out of acceptable range. However, study should be conducted to establish an optimal balance between retraining an algorithm with genuine and imposter samples.

#### 6.2.5. Adaptive Threshold

Instead of updating the keystroke reference template, [35] proposed to readjust the matching threshold. This method circumvents the complexity of retraining sample data over potentially complex algorithm. In [39], threshold is repeatedly reassessed upon every successful authenticated user access. Users are also given two trials to be validated, with the assumption that legitimate users are more likely to pass an authentication test; this ends up with high adaptation accuracy.

### 6.3. Outlier Handling

Outlier is an atypical or extreme data that is significantly out of norm. For instance, a keystroke timing value of 3000 ms would likely be considered as outlier, since the mean range of probable human keystroke timing value is between 96 to 825 ms [39]. The origin of noise in data may be initiated by random pauses or hesitations [61] or physical state of user or environmental condition [38] that disturbs user typing and could skew the feature measurements [77]. Such outlier, if not specially handled, may affect classification outcome and consequently degrades system performance. Noise removal [110], data cleaning [111], or extreme outlier removal [39] might lead to better performance as claimed by [4, 77, 133].

Several methods of outlier handling exist in the literature, where an adjustable constant is the most common [38, 59, 75, 113]. The following inequality describes the elimination condition:

(7) $$\begin{array}{c} x\leq c\cdot \sigma , \end{array}$$

where *x* refers to a timing value instance, *c* represents an adjustable constant, and *σ* is the standard deviation of a reference template. Timing value *x* will be removed if (7) is not met. A large value of *c* indicates that more timing value will be discarded as training sample or may also imply that a user did not type consistently [20]. Nevertheless, precaution should be taken during the establishment of discarding threshold so that the remaining number of samples is not too small for training.

 Another similar approach taken by [84, 109] is by removing the outlier if any of the value deviated from the upper or lower of a predetermined percentage (e.g., 10%). Kaneko et al. [34] used an empirical fixed value (e.g., 240 ms) as determination criteria on detecting noisy data. This method might not be scalable since outlier is dissimilar for different individuals due to diverse typing proficiency. Other methods such as f-distribution [4] and principle component analysis [32] have also been explored. 

 Human judgement on inconsistency of data is subjective and may be dissimilar among different persons [111]. Furthermore, manual outlier detection and removal is infeasible in an automated system. Thus, [110] proposed using Genetic Algorithm-Support Vector Machine that can automatically select the relevant subset of feature and disregard noisy data without human intervention. Although evidences in the literatures show that removal of outlier generally results in better performance, it may reduce training data samples. As compensation, significant effort has to be put to collect larger data sample.

### 6.4. Fusion and Multimodal

Multimodal biometrics fusion has been widely adopted and well known for its ability to improve the overall performance of a biometrics system [143–145]. This is made possible as fusion utilizes information from more than one source or feature data. The extra information generated by this additional layer aids in better discrimination of imposter from genuine user.

#### 6.4.1. Feature Fusion

The combination of different variants of keystroke feature data is one of the most common fusion methods employed. For example, [93] concatenated four different keystroke durations and latencies forming a large timing vector instead of using them individually. On the other hand, [37] merged user typing pressure information and traditional keystroke timing data and obtained a better result as compared to using them separately. 

#### 6.4.2. Score Fusion

Score level fusion combines output scores from different classifiers prior to decision making. Since output scores generated from different classifiers may not always be in a unified range; therefore, it is essential to normalize the scores before fusion [146]. A commonly used normalization method includes maximum difference between score, z-score, tanh-estimator, and double sigmoid [28]. However, not all score level fusions require prior normalization. For instance in experiment [18], score produced by Gaussian probability density function and direction similarity measure are both readily within the same range of 0 and 1; hence, normalization is unnecessary. Combining scores from different matchers usually involves fusion rules. Simple and common rules found in the literature include weighted sum [147], maximum and minimum score selection, median, product rule, and sum rule [146].

#### 6.4.3. Decision Fusion

Fusion at decision level is among the simplest fusion scheme available since it has the benefit of not requiring any change to the internal structure of other modularity. Scores produced by different classification algorithms are compared against authentication threshold and generates individual preliminary authentication decision. Final decision is obtained by voting schemes such as majority [37], AND, and OR voting [148].

#### 6.4.4. Multilayer Fusion

It is believed that as more information were combined, genuine and imposter distinction could be attained at a higher probability [3]. The authors proposed a two-layer fusion framework that not only merges information from different keystroke features but also matching scores from two detectors. Experimental result strongly supports the advantage of information fusion.

#### 6.4.5. Multiple Biometric Modality Fusion

Keystroke dynamics may not be sufficient to be a sole authenticator due to the rather low accuracy as compared to established biometrics such as fingerprint and iris modality. Therefore, researchers have tried to combine multiple biometrics with the objective to make it harder for an intruder to spoof several biometric traits simultaneously. A multibiometrics application system has been proposed by [56] utilizing keystroke dynamics and fingerprint feature. Aside from a match fingerprint minutiae data, input pattern of PIN number must also correspond to a certain similarity, thus, doubling up the authentication criteria. On the other hand, [149] proposed the fusion of keystroke input and unique click pattern on a Knock Pad as authentication feature, which reduced the need of relying on long and complicated password. Experimental result by [150] suggested that the combination of keystroke dynamics and face recognition was able to obtain better result than employing each trait independently. 

### 6.5. Keystroke Dynamics Quality Measure and Control

When it comes to performance enhancement strategy, a lot of research works have been focusing on improving classification algorithms. However, [108] suggested that quality measure of keystroke patterns is a much more determinant criteria than classifier employed. Quality of user template has a direct impact on the performance of an authentication system [38]; hence, designing a good and discriminative keystroke feature profile is a crucial process that should not be undermined.

#### 6.5.1. Timing Resolution

One of the major factors that contribute to system performance is timing resolution, and thus suitable timing resolution is important so that the keystroke timing vector generated can characterize user typing cadence in the right precision. 

Earlier research work was implemented at a timing resolution of 10 ms [43, 151], unfortunately detector performance could be limited by the use of such low resolution clock [125]. However, due to computer processing capacity at that point of time, this was the best precision achievable. Today, high performance computer can reach a clock resolution of micro or even nanoseconds easily. Although greater timing resolution is able to increase performance [152], precision as high as nanoseconds is not necessary since no one can achieve such a fast typing speed. Ever since, the most widely used resolution was in the range of 0.1 [18, 33, 103] to 1 ms [23, 42, 57]. It was recommended by [39] that a resolution of at least 1 ms should be used to capture keystroke events.

Reference [125] was dedicated on discussing the relation of clock resolution and the performance of keystroke dynamics detectors. The authors evaluated three established detectors against different clock resolution precisions. Experimental result showed that there is performance improvement, albeit small, by using high-resolution clock as compared to lower ones.

#### 6.5.2. Artificial Rhythm and Cue

The quality of keystroke dynamics can be improved artificially by increasing the distinctiveness and peculiarity of typing pattern [153], thereby evades the increase of hardware implementation cost.

Uniqueness and consistency are the two core factors revealed by [154], which determine quality of keystroke feature. Uniqueness associated with how dissimilar an intruder's input pattern compared to the reference template while consistency implies to what degree a user's typing pattern matches the enrolled template during registration. The author proposed that uniqueness could be enhanced by incorporating artificially designed rhythms during input process such as pauses, musical rhythm, staccato, legato, and slow tempo. Similarly, auditory and visual cues were introduced with the aim of increasing consistency. As a consequence, legitimate users' typing patterns could be better separated from intruders' [108].

By having better quality data, the number of enrolment samples required for constructing reliable reference template can be radically reduced [29]. Thus, using artificial rhythms and cues has an additional advantage of reducing user's burden in terms of providing repeated samples during registration stage.

#### 6.5.3. Keyboard Partitioning

An alternative way of increasing the quality of keystroke feature is to increase the complexity and variety of input data. Magãlhaes et al. [116] proposed to divide keyboard into four disjoint zones, forcing user to choose characters scattered across the keyboard. It was reported that the best result could be achieved when user did not type at their maximum speed. Since keyboard partitioning is able to slow down user typing speed, eventually provides more accuracy to keystroke dynamics recognition system.

However, the obvious disadvantage is the restricted password selection choice that is imposed from the added requirement to select characters from four different keyboard regions. Nevertheless, it was argued by the author that this was a small price to pay for security, especially for critical e-commerce sites.

#### 6.5.4. Length of Input

Researcher has also argued that a longer string as input is the key to improve the performance [21]. Investigation has been conducted to determine the most appropriate string length for authentication accuracy. Results suggested that the best performance was achieved at the string length of 13 to 15 characters [92]. Although the result in the experiment conducted was not exceptional, but it shows sign of improvement as string length increases. Therefore, string size should be an essential consideration for future research work on keystroke dynamics.

## 7. Result Discussion

Since it is impossible to compile every single research study, we will divide them into a few categories for discussion. These categories encompass static and dynamic authentication modes, pressure-based, mobile, and numerical input experiments. 

### 7.1. Static Authentication Mode

Both dwell time (DT) and flight time (FT) are often extracted as feature vector for static authentication. There was no clear comparison made on which timing vector performed the best; however, [3] suggested that the combination of both DT and FT produced a better result than using them independently. The best combination of keystroke features and methods yield a respectable EER of 1.401%. 

By far the experiment that involved the largest number of participants was conducted by [67]. A whooping 1254 users were involved, although only half of that amount completed the whole data collection process. Experiment with around 100 users is considered moderate in keystroke dynamics domain thus far as seen in Tables 4 and 5.

By using an autoassociative multilayer perceptron algorithm and support vector machine as novelty detector, [84] was able to attain impressive result of nearly 0% for both FAR and FRR. In spite of good performance, users were required to repeatedly provide their password 150 to 400 times, which may not be feasible in real world situation. Furthermore keystroke samples at the later repetition may be significantly different from the initial few ones as user gets accustom to the input text. Therefore, the best practice would be to perform data collection over a few sittings. At such, user will not be burdened by large repetitive inputs and the keystroke feature captured reflects the gradual change in typing pattern due to familiarization over time. For example in [93], data collection was scattered across five sessions separated by one week apart with 12 repetitions of input samples per session.

Another interesting experimental variable is the degree of freedom user is given during data collection phase. Numerous research works confined user to a predefined input text and yet yielded reasonable performance (EER < 5%) such as [3, 21, 42]. These results may be improved further, in particular FRR, if users are allowed to choose their own favourable string. The argument here is that familiarity of a certain string will most likely promote consistency, thereby reducing intraclass variability. Therefore, if an experiment consists of both fixed string and user selected string, comparison between the effects of input string selection can be deduced. 

Similarly, the effect of user typing on a familiar verses prearranged device may cast some significance to the recognition performance. Although it may not be entirely possible to provide such flexibility due to various reasons and constrains, it is seen as a potential consideration in terms of experimental design for future research work.

### 7.2. Dynamic Authentication Mode

In an effort toward developing a robust online examination authentication system, [75] investigated the use of not only keystroke feature but also stylometry. Stylometry was known as the study of recognizing authorship from the linguistic styles of an author. The *k*-nearest neighbour classifier has been applied. The experimental result shows that performance of traditional keystroke feature is superior to stylometry. This may be due to the operation of stylometry that depends heavily on words and syntax-level units; therefore, much longer text inputs are required for better recognition. 

Since dynamic authentication mode requires large amount of input text, [76] tried to utilized *n*-graph feature timing vector instead of di-graph. A fairly straight forward displacement of each *n*-graph sample pair of words are computed for distance measurement. One of the challenging scenarios of using *n*-graphs in free text input is the need to collect the same *n*-graphs for comparison. The flexibility of input text is essential to dynamic authentication. The immediate solution will be to gather as much typing inputs as possible, which translates into longer waiting time to collect enough keystrokes before authentication can effectively takes place. This might be the reason why majority of experiments have chosen di-graph for feature vector construction as shown in Table 6. This observation was supported by [59], where the experiments have been restricted to digraphs, tri-graphs, and four-graphs due to relative limited number of shared samples. On a side note, the author also pointed out that comparing sample over different typing languages will be possible provided the two languages shared same legal *n*-graphs.

A different approach was employed by [124], whereby a set of fixed frequent appearing English words was used to form the basis of user typing reference template. At such, the wait for a word pattern to appear can be reduced whilst exploiting the stability of fixed text authentication in a free text environment. 

Due to the popularity of communication technologies such as instant messaging, online social networks chatting, and text messaging, the usage of non-English sequences (short hand notation and informal English abbreviations) has been increasingly dominance [124]. The research work proposed a goodness measure to quantify the quality of a series of fixed text based on the criteria of accuracy, availability, and universality of the text sequence. The author found that non-English words were more accurate than English words in classification. This is an interesting preliminary finding that should be utilized for future study on different languages such as Italian, Korean, and Chinese.

### 7.3. Keystroke Pressure Feature

Keystroke pressure feature has been overlooked mainly due to the need of special input devices as in Table 7. Remarkable result (EER ≤ 1%) has been obtained by [48, 51, 158]; however, the number of subjects involved was too small (less than 10) to draw a strong conclusion. Conversely, although [52] reported a poorer result but 100 users participated in the experiment that might better reflect the scalability of the proposed method. By far the experiment that involved the largest test samples and yet achieved encouraging result (EER = 1.4) is [37]. The author constructed a feature vector that not only consisted of traditional timing vector but also the extraction of five global pressure attributes. Dynamic time warping, which has been commonly employed in speech and signature recognition was used to calculate the distance between pressure sequences.

It is worth noticing that [102] demonstrated a very unique way of extracting keystroke pressure. The author proposed an indirect method to detect key-typed forces by analyzing sound signals generated upon striking on the keyboard with a sound recorder. Although without the need of pressure sensors attached to the keyboard would be an added advantage, the susceptibility to environmental noise may deter the quality of feature captured. 

 By far none of the experiments utilized pressure sensitive screen on mobile device. Since we are stepping into the post-pc era, smart phones and high-end tablet devices are commonly built-in with accurate pressure sensitive screens. It will be interesting to see how future research work corresponds with keystroke pressure feature by fully exploiting this readily available hardware technology. 

### 7.4. Mobile Platform

A handful of research works have identified the potential of mobile devices and tried to integrate keystroke dynamics recognition in the mobile platform as shown in Table 8.

The earliest keystroke dynamics research performed entirely in mobile devices was [50] in year 2007. The experiment attempted to authenticate user by monitoring user routine interaction on the mobile phone such as entering telephone number and text messaging. Feed forward multilayered perceptron (MLP) has been used to model user keystroke activity. However, the extra computational power required to run the MLP was a great concern for mobile devices at that time. It is awaited to be seen if the computational time could be lower with such algorithm performed on modern devices. Since then more commercial devices have been used as experimental platform. For instance, [50] required user to input 4 digit PIN number on a Samsung SCH-V740 mobile phone via a customized prototype software. Despite the short number of input, an EER of 13% was achieved and further enhanced to 4% after the introduction of artificial rhythm and cues.

By far there was no research work performed on a more recent smart phone platform such as iphone and android. These devices are more commonly available in the market for the coming years and have superior processing capability as well as various sensors such as pressure sensor, gyroscope, and accelerometer. These sensors may have the potential to bring an extra dimension to keystroke feature and thus enhancing the overall quality and uniqueness. 

### 7.5. Numerical Data Input

As discussed in earlier section, previous studies suggested that complexity and length of input show a direct relationship with the proficiency of keystroke dynamics recognition. Input device such as those embedded in ATM machine, access control panel, and card payment machine do not have the luxury of alphabetic input. Therefore, the ability to select complex secret phrase combination will significantly be limited. Moreover, such input devices usually require only 4 (credit or debit card PIN) to 10 (numeric PIN code) length of numeric digits. Thus, it is interesting to see how keystroke dynamics recognition performs exclusively with numerical inputs.  Table 9 lists a summary of research works on keystroke numeric inputs.

A keypad that looked and felt exactly as the one that was deployed on commercial ATM machines has been adapted by [26]. Euclidean distance measure was used to calculate the difference between test vectors. A remarkable FRR of 0% was achieved at 15% for FAR.

On the other hand, [24] abandoned physical keyboard by introducing four pairs of infrared sensors to project a virtual numeric grid keyboard. In the experiment, user's finger had to be held at a 90 degree angle to the surface of the keyboard. A 78–99% classification accuracy was reported by using *k*-nearest neighbor classifier and multilayer perceptron. The feasibility of the sensor keyboard in real life has been called into question. We could not make a clear cut conclusion if a greater length of digit produces better result due to the small difference between the length of inputs. Hence, experiment on longer numeric length (e.g., 16 digits) that bears a resemblance to credit or debit card number should be investigated. 

### 7.6. Commercialized Software

A handful of commercialized software is available in the market such as Biopassword [161], TypeSense [162], and AuthenWare [163]. Regrettably the effectiveness and methodology are not publically available due to copyright issues; therefore, it is difficult to evaluate the effectiveness of each system.

## 8. Opportunity

*Future Research Opportunities and Recommendations*. After reviewing the keystroke dynamics literature studies, below are some of the suggestions and potential areas that can be explored by researchers in the keystroke dynamics domain.

### 8.1. Feature Quality Measure and Enhancement

One of the immediate approaches to enhance performance of keystroke dynamics recognition is by focusing on introducing new detector or classification algorithm. However, another potential route that may be looked into is by providing these detectors with higher quality feature data. A bold approach taken by [154], which introduced the use of artificial rhythm and cues to increase uniqueness of typing feature is a preliminary step forward in this aspect. Feature quality may also be boosted by fine tuning timing resolution, dynamic feature selection, data filtration, and feature data fusion. 

### 8.2. Mobile Platform and Touch Screen Devices

As technology evolution grows, mobile and portable devices have been ubiquitous in human's daily life. Smart phone and tablet have ever increasing memory and processing power as compared to few years ago. Furthermore, the introduction of advance and sensitive miniature hardware sensors such as multitouch screen, pressure sensitive panels, accelerometer, and gyroscope has the potential of unleashing new feature data. This improved hardware is now readily available and paves a way for future keystroke dynamics research study on this platform. 

### 8.3. Dynamic Authentication

As compared to static one-off authentication mode, keystroke dynamics research on dynamic or continuous authentication is still rather inadequate. Several research works in the literature have laid the foundation on continuous authentication on free and long text input. Potential untapped area would be continuous authentication on foreign languages such as Korean, Chinese, Italian, and non-English word (informal short abbreviation). Additionally, experimental platform should be accentuated on web browser-based authentication since the computer usage trend has be shifted from operating system-based application to browser-based cloud services. Therefore, continuous and uninterrupted validation of user identity throughout the session of accessing these services within the online platform is in high demand. 

### 8.4. Retraining Mechanism Evaluation

Keystroke dynamics biometrics are subdomain of behavioral biometrics that have the possibility of evolvement over time. More extensive studies need to be conducted particularly on update mechanism if keystroke dynamics are to be used as a long-term security enhancement tool. Result evaluation and the effectiveness of a retraining algorithm or framework should be assessed in stages across a longer period of time (e.g., 6–12 months) to allow time for accommodating the gradual change of typing pattern.

### 8.5. Benchmark Dataset

In long term, keystroke dynamics research community should be encouraged to come up with a shared benchmark dataset wherever possible. Development of homemade dataset may cater to individual experimental needs; however, experiment result cross-comparison between different methodologies employed may not be conclusive. Furthermore, some researchers may not have the resource to develop a proper dataset for experiment. We would recommend the community to produce 3 types of dataset with both free and fixed text from keyboard input as well as numerical input data from mobile phone. These would be sufficient to cater keystroke dynamics research across the 3 major platforms. A sample size of at least 100 or more should be an initial aim. Dataset owner is encouraged to share the data collection tool if possible, so that others may help contribute to the data collection process. At such, not only can the benchmark sample size increases gradually over time but also the opportunity to collect keystroke typing samples from diverse communities across the globe.

## 9. Conclusion

Majority of the keystroke dynamics research works from the last three decades have been summarized and analyzed in this paper. It is by no means to be an exhausted archive of all research works in the keystroke dynamics domain, but it was collected with the resource available and to the best of our knowledge at the point of writing. The aim of this review paper is to provide a reference for researchers to further look into others work to identify promising research direction for further study. We believe that this will also significantly lower the entry barrier especially for novice researchers who are interested in keystroke dynamics.

The literature study suggested that keystroke dynamics biometrics are unlikely to replace existing knowledge-based authentication entirely and it is also not robust enough to be a sole biometric authenticator. However, the advantage of keystroke dynamics is indisputable such as the ability to operate in stealth mode, low implementation cost, high user acceptance, and ease of integration to existing security systems. These create the basis of a potentially effective way of enhancing overall security rating by playing a significant role in part of a larger multifactor authentication mechanism.

## Figures and Tables

**Table 1:** Overview of different authentication approaches.

Approach	Advantage	Disadvantage	Example
Knowledge	Effortless High acceptance	Forgotten Shoulder spoofing	Password PIN

Token	Cheap Simple deployment	Lost and theft	Smart card Minidevices

Biometrics	Deter sharing Unique Unforgettable	Cost Invasive	Fingerprint Voice Keystroke

**Table 2:** Comparison with existing survey paper on keystroke dynamics.

Paper	Number of reference cited	Latest reference
[6]	25	2004
[7]	35	2009
[8]	72	2009
This paper	163	2012

**Table 3:** Comparison between different public data sets.

Feature	[97]	[98]	[99]
	Timing	Timing and pressure	Timing
Data size	100	104	51
Input	greyc laboratory	pr7q1z Jeffery Allen drizzle	.tie5Roanl
Scope	Genuine only	Genuine (7), imposter (97)	Genuine only
Repetition	6	89–504 (genuine), 3–15 (imposter)	50
Interval	1 or 2 sessions per week	—	At least 1 day apart each session
Session	5	—	8

**Table 4:** Comparison between research works with static authentication mode (short inputs).

Study	Data size	Latency	Input repetition	Input freedom	Method	FAR (%)	FRR (%)	EER (%)	Input sessions	Device freedom
[113]	33	FT	8	Yes	Mean, standard deviation	0.25	16.36	—	—	No
[61]	26	FT	30	Yes	Bayesian and minimum distance classifier	2.8	8.1	—	Once	No
[155]	24	FT	2	Yes	Perceptron algorithm	8	9	—	Yes	No
[9]	15	DT, FT	—	Yes	ART-2, RBFN, and LVQ	—	—	0	Yes	No
[103]	10	DT, FT	20	Yes	Inductive learning classifier	9	10	—	—	No
[84]	21	DT, FT	150–400	Yes	Autoassociative multilayer perceptron, SVM	0	0.814	—	—	—
[134]	100	DT	100	No	Genetic algorithm	—	—	95*	Yes	—
[21]	41	DT, FT	5	No	Random forest decision tree	—	—	2	Yes	Yes
[42]	30	DT, FT	10	No	Sequence alignment algorithms	0.15	0.2	0.35	Yes	—
[17]	21	DT, FT	—	Yes	K-means, euclidian	—	—	3.8	Yes	—
[86]	100	DT, FT	—	No	Multilayer perceptron	1	8	—	Once	—
[39]	41	DT, FT	30	Yes	Gaussian mixture modeling	4.3	4.8	4.4	Yes	Yes
[89]	100	DT, FT	6	No	Bayesian, Euclidean, hamming distance	—	—	6.96	Yes	No
[67]	1254	DT, FT	20	No	Mean, standard deviation	16	1	—	Once	Yes
[96]	16	DT, FT	5	No	Bayesian, Euclidean	—	—	4.28	Yes	—
[108]	25	DT, FT	30	Yes	Gauss, Parzen, K-NN, K-mean	—	—	1	—	—
[123]	51	DT, FT	50	No	Manhattan distance	—	—	7.1	Yes	No
[93]	100	DT, FT	12	No	Support vector machine	—	—	15.28	Yes	No
[3]	100	DT, FT	10	No	Gaussian PDF, direction similarity measure	—	—	1.401	Once	Yes
[138]	117	DT, FT	5	Yes	Support vector machine	—	—	11.83	Once	No

∗ Indicates performance measurement in terms of accuracy, similar but inverse to EER where value closer to 100% indicates better performance.

**Table 5:** Comparison between research works with static authentication mode (long inputs).

Study	Data size	Latency	Input repetition	Input freedom	Method	FAR (%)	FRR (%)	EER (%)	Input sessions	Device freedom
[12]	7	FT	1	No	*t*-Test	—	—	95^†^	Yes	No
[40]	43	DT, FT	9	No	Parallel decision trees, Monte Carlo	0.88	9.62	—	Yes	—
[156]	31	FT	2	Yes	Degree of Disorder	1.99	2.42	—	—	No
[87]	—	DT, FT	—	No	Decision tree c4.5, j48	—	—	93.3*	—	—
[77]	118	DT, FT	5	Yes	Euclidean distance	—	—	97.9*	—	Both
[135]	24	DT, FT	60	Yes	Support vector machine	0.76	0.81	1.57	—	—
[88]	112	DT, FT	—	No	Weighted Euclidean distance	—	—	100*	Yes	—
[157]	35	FT	—	No	Kolmogorov-Smirnov test	—	—	7.55	—	No
[34]	51	FT	5	No	Euclidean distance	—	—	0.84	No	—
[115]	33	DT, FT	9	No	Naive Bayesian	—	—	1.72	Yes	Yes
[65]	189	DT, FT	—	No	Weighted Euclidean distance, array disorder	0.01	3	—	No	—
[121]	20	FT	5	Yes	Euclidean distance	2	4	—	No	—
[126]	50	FT	—	Yes	Degree of Disorder	—	—	10	Yes	Yes

∗ Indicates performance measurement in terms of accuracy, similar but inverse to EER where value closer to 100% indicates better performance.

† Indicates confidence interval, similar to accuracy.

**Table 6:** Comparison between research works in dynamic authentication mode.

Study	Data size	Maximum number of graphs	Freedom of input	Method	FAR (%)	FRR (%)	EER (%)	Platform
[20]	31	di-graph	Yes	Weighted mean, Standard deviation	—	—	90*	OS
[58]	63	di-graph	Yes	*k*-nearest neighbor	—	—	83.22–92.14*	OS
[59]	205	*n*-graph	Yes	Degree of disorder, mean, standard deviation	0.5	5	—	Web
[124]	22	di-graph	Yes	Bhattacharyya distance, goodness measure	—	—	86.47*	OS
[74]	61	*n*-graph	No	SVM and decision tree J48	14.5	1.78	—	OS
[105]	21	di-graph	Yes	Degree of disorder and histogram-based Density estimation	0.14	1.59	—	OS
[72]	10	di-graph	—	Random forest decision tree	0.41	0.63	0.53	—
[107]	21	di-graph	Yes	Random forest decision tree	3.47	0	1.73	Web
[75]	30	di-graph	No	*k*-nearest neighbor	—	—	0.5	Web
[22]	55	*n*-graph	Yes	Spearman's foot-rule distance-metric	2.02	1.84	—	Web
[76]	186	di-graph	Yes	Degree of disorder	1.65	2.75	—	Web

∗ Indicates performance measurement in terms of accuracy, similar but inverse to EER where value closer to 100% indicates better performance.

**Table 7:** Comparison between research works involving keystroke pressure feature.

Study	Data size	Method	FAR (%)	FRR (%)	EER (%)	Equipment customization requirement
[159]	10	Adaptive neural fuzzy inference system	2.3	25.2	—	Yes
[25]	9	ANOVA	—	—	2.4	Yes
[23]	—	Fuzzy ARTMAP	0.87	4.4	—	Yes
[37]	100	Dynamic time warping	1.4	1.4	1.4	—
[137]	5	Support vector machine	0.95	5.6	—	Yes
[52]	100	ARTMAP-FD	—	—	11.78	Yes
[48]	10	*k*-nearest neighbor	—	—	1	—
[26]	30	Euclidean distance	15	0	10	Yes
[158]	10	Probabilistic neural network	—	—	1	Yes
[49]	30	Radial basis function network	2	0	—	Yes
[51]	7	Multilayer feed-forward network	0	0	—	Yes
[102]	20	Fast artificial neural network	4.12	5.55	—	No

**Table 8:** Comparison between research works performed on mobile platform.

Study	Data size	Text	Digit	Method	FAR (%)	FRR (%)	EER (%)	Input device
[50]	32	Yes	Yes	Feed-forward multilayered perceptrons	—	—	12.8	Modified Nokia 5110
[160]	3	No	Yes	Mean, standard deviation	—	—	90*	Modified Renesas H8S-2377
[28]	30	Yes	No	Mean, standard deviation	—	—	13	Nokia 6608
[29]	25	No	Yes	—	—	—	4	SAMSUNG SCH-V740
[55]	25	Yes	No	Fuzzy classifier	2	0	—	Symbian smart phone

∗ Indicates performance measurement in terms of accuracy, similar but inverse to EER where value closer to 100% indicates better performance.

**Table 9:** Comparison between research works based on numerical inputs.

Study	Data size	Digit length	Method	FAR (%)	FRR (%)	EER (%)	Input device
[24]	7	—	*k*-nearest neighbor and multilayer perceptron	—	—	78–99*	Infrared virtual num-pad
[25]	9	4	ANOVA	—	—	2.4	Customized num-pad
[82]	20	8	Hidden Markov model	—	—	3.6	Normal keyboard
[137]	5	6	Support vector machine	0.95	5.6	—	Modified keyboard
[48]	10	10	*k*-nearest neighbor	—	—	1	Notebook touch pad
[26]	30	4	Euclidean distance	15	0	10	Modified ATM num-pad
[158]	10	10	Probabilistic neural network	—	—	1	Notebook touch pad
[33]	28	10	Random forest decision tree	0.03	1.51	1	Apple keyboard
[47]	25	10	Back propagation neural network	—	—	94.8*	Normal keyboard
[4]	24	4	Support vector machine	—	—	2	Modified Microsoft keyboard

∗ Indicates performance measurement in terms of accuracy, similar but inverse to EER where value closer to 100% indicates better performance.
